# Oridonin supplementation attenuates atherosclerosis via NLRP-3 inflammasome pathway suppression

**DOI:** 10.25122/jml-2022-0292

**Published:** 2023-07

**Authors:** Ahmed Sultan, Bassim Mohammad, Najah Rayish Hadi

**Affiliations:** 1 Department of Pharmacology and Therapeutics, College of Pharmacy, University of Al-Qadisiyah, Al Diwaniyah, Iraq; 2 Department of Pharmacology and Therapeutics, College of Medicine, University of Al-Qadisiyah, Al Diwaniyah, Iraq; 3 Department of Pharmacology & Therapeutics, Faculty of Medicine, University of Kufa, Kufa, Iraq

**Keywords:** Oridonin, atherosclerosis, NLRP-3 inflammasome

## Abstract

Atherosclerosis, a long-term inflammatory and immune condition affecting medium- and large-sized arteries, results in the thickening of artery walls and the accumulation of inflammatory cells and fatty streaks that establish fibrous capsules with macrophages at the site of injury. Atherosclerosis has a major impact on the pathogenesis of cardiovascular diseases. Oridonin has been shown to exclusively inhibit the NLRP3 inflammasome without affecting the activation of AIM-2 or NLRC-4 inflammasomes. The current study aimed to evaluate how adding Oridonin to a diet impacts the onset of atherosclerosis. Twenty-one male rabbits weighing 1.5 to 2.0 kg were included in the study. The rabbits were kept in controlled environmental conditions and divided into three groups: a normal control group fed a conventional chow diet, an atherogenic control group fed a high-cholesterol diet (2% cholesterol-rich), and an Oridonin-treated group (Ori) fed an atherogenic diet supplemented with Oridonin (20 mg/kg) administered orally once daily. Compared to animals on a normal diet, an atherogenic diet was associated with a statistically significant (p=0.001) increase in the mean expression of the NLRP3 inflammasome mRNA. The Oridonin-treated group showed a statistically significant (p=0.001) decline in the mean expression of NLRP3 inflammasome mRNA compared to the atherogenic group. Furthermore, the initial atherosclerotic lesion in the group treated with Oridonin was statistically (p=0.001) less severe compared to the atherogenic group. Finally, Ori treated group had significantly (p≤0.001) lower IL-1B immunostaining intensity than the atherogenic group (mean rank 14.5,25 respectively). The study concluded that Oridonin supplementation resulted in less severe initial atherosclerotic lesions, likely due to the suppression of NLRP3 inflammasome and the anti-inflammatory effect through the downregulation of IL1B expression.

## INTRODUCTION

Atherosclerosis, a long-term inflammatory and immune condition affecting medium- and large-sized arteries, results in the thickening of artery walls and the accumulation of inflammatory cells and fatty streaks that establish fibrous capsules with macrophages at the site of injury. This pathological process has a significant impact on the pathogenesis of cardiovascular diseases (CVD) [1]. Naturally, the buildup of lipids in an interstitial space requires a source of lipids. Circulating lipid, which is supposed to be involved in important physiological processes, is afterward entrapped in the tissue of the blood artery and never reaches the intended target cells. Greater blood lipid levels, particularly higher cholesterol levels, seem to promote this accumulation [2, 3]. Low-density lipoprotein (LDL) particles often undergo chemical modifications, making them susceptible to ingestion by macrophages [4]. Monocytes undergo maturation into macrophages, essential for the inflammatory response, as soon as they enter the vascular wall. Although there is clear variation in plaque macrophages, recent studies have explored how the bulk of these cells are conventionally rather than alternatively activated [5-7]. Several clinical and experimental studies have revealed that IL-1 is an important pro-atherogenic cytokine for the progression of atherosclerosis [8-10]. These results showed that the progression of atherosclerosis could be facilitated by NLRP3 inflammasome. In 2010, Duewell *et al*. [11] made the initial case for the importance of NLRP-3 inflammasome in the progression of atherosclerosis. They focused on cholesterol crystals as the source of DAMPs and found that these crystals substantially activated NLRP-3 inflammasome in macrophages. Additionally, they showed that the presence of oxidized LDL, which has a high atherogenic potential, could induce cholesterol to crystallize and can activate priming signals that result in the expression of NLRP-3 and proIL-1, suggesting that oxidized LDL may trigger signals 1 and 2, leading to the release of IL-1 [11]. A subsequent study by Sheedy *et al*. [12] found that incorporating oxidized LDL via the scavenger receptor, CD36 led to intracellular cholesterol crystallization. These data, along with the fact that ApoE-/- mice, a commonly used atherosclerosis-prone animal, had less severe atherosclerosis when IL-1 was absent, suggested that NLRP3 inflammasome-driven IL-1 production aided in the development of atherosclerosis [8, 12]. The herb plant *Rabdosia rubescens* has a bioactive ent-kaurane diterpenoid called Oridonin, which is widely used in traditional Chinese medicine [13]. According to one study [14], Oridonin (Ori) constitutes a covalent connection with cysteine 279 of the NACHT domain of NLRP3, eliminates the contact between NEK-7 and NLRP3, and suppresses NLRP3 inflammasome subsequent stimulation. Oridonin exclusively inhibits the NLRP3 inflammasome, and it has no inhibitory effects on the activation of the AIM-2 or NLRC-4 inflammasome. Ori showed notable curative and preventative benefits in mouse models of T2-D, peritonitis, and gouty arthritis [14]. The aim of this study was to investigate the role of Oridonin, an NLRP3 inflammasome inhibitor, in the progression of atherosclerosis.

## MATERIAL AND METHODS

### Animals preparation

Twenty-one male rabbits weighing 1.5 to 2.0 kg were included in this study. All experimental procedures followed the guidelines at the Department of Pharmacology, Faculty of Medicine, University of Kufa, for the ethical care and use of laboratory animals in scientific research. The animals were kept at 25 degrees Celsius, 45 percent humidity, and a 12:12 h light:dark cycle. Before the studies, they were given two weeks to acclimate while being fed standard laboratory chow and provided unlimited access to water.

### Study design

Following a 2-week acclimation period, animals were divided into 3 groups, each consisting of seven rabbits.

1. Normal control group: Rabbits in this group were fed a conventional chow diet and had access to tap water throughout the 8-week trial period.
2. Atherogenic control group (induced untreated group): Rabbits in this group were fed a high-cholesterol diet containing 2% cholesterol.
3. Oridonin-treated group (Ori): Rabbits in this group were fed an atherogenic diet (enhanced with 2% cholesterol) along with Oridonin powder, which was dissolved in water and administered once daily via the oral route at a dose of 20 mg/kg. Throughout the 8-week research period, the medication and atherogenic diet were sustained.

### Animal model of atherosclerosis

To induce atherosclerosis, the rabbits in the atherogenic control and Oridonin-treated groups were fed a high cholesterol diet (2% cholesterol, BDH Chemicals Ltd Poole England, prod 43011) for eight weeks, leading to atherosclerosis progression [15].

### Sample preparation and collection

At the end of the 8-week study period, the rabbits were fasted for 16–18 hours and anesthetized with intramuscular ketamine (66 mg/kg) and xylazine (6 mg/kg) [16]. The thoracotomy was used to open the chest, a direct blood sample was collected from the heart, and the aorta was divided. Then the following tests were run:

1. Evaluation of the NLRP3-inflammasome maker using RT-PCR.
2. Histopathological investigation of the aorta to detect atherosclerosis and IL-1B immunostaining.

### Real-Time PCR Quantitative (qPCR)

Real-Time PCR was used to quantitatively analyze the expression of the target gene (NLRP3 inflammasome), normalized by the housekeeping gene (GAPDH), in blood samples from the study groups. Total RNA was extracted from aortic tissue samples using the TRIzol^®^ reagent kit, following the manufacturer's guidelines. Two quality checks on the extracted RNA were carried out using the NanoDrop spectrophotometer (THERMO, USA) to evaluate and measure the extracted total RNA. The first step was to calculate the RNA concentration (ng/L), and the second was to calculate the RNA purity by measuring the absorbance in a spectrophotometer at 260 and 280 nm on the same NanoDrop equipment. According to the procedure outlined by Promega Company, USA guidelines, extracted RNA was processed with the DNase I enzyme to eliminate the minute quantities of genomic DNA from the eluted total RNA. As directed by the manufacturer, the M-MLV Reverse Transcriptase kit was used to create cDNA from DNase-I treated RNA samples for the Pten and GAPDH genes.

The primers for target genes (NLRP3) and housekeeping gene (GAPDH) were designed using the NCBI-Gene Bank database and Primer 3 design online. These primers were provided by Bioneer Company, Korea (Table 1).

**Table 1:** Primers used in the study

Primer	Sequence (5’-3’)		Product Size	Genbank
NLRP3	F	TCGTTGTGCGTTTCCTCTTC	133bp	XM_017339176.1
	R	TTGGCGTTGGCTTTTGTCTC		
GAPDH	F	GTCAAGGCTGAGAACGGGAA	95bp	NM_001082253.1
	R	CCAGCATCACCCCACTTGAT		

### Tissue sample preparation

Aortic arches were externalized, cleared of connective tissue and adhering fat, and divided into two pieces. One piece of aortic tissue was immediately immersed in a 10% formaldehyde solution for 24 hours to create histopathology and immunostaining slides. The second piece of aortic tissue was homogenized in TRIzol^®^ reagent to assess the NLRP3 inflammasome marker using the RT-PCR method.

### Immunostaining for IL-1B (Novus Biologicals, Cat NO: NB600-633)

Immunostaining was performed according to the supplier's instructions (Novus Biologicals, Cat NO: NB600-633). Semi-quantification of antigen expression was evaluated under a light microscope at 40X magnification. Staining intensity was scored by two pathologists as follows: 0 indicated no staining, 1 indicated weak staining, 2 indicated moderate staining, 3 indicated strong staining, and 4 indicated very strong staining.

### Statistical analysis

Data analysis was performed using SPSS version 26. The LSD post-hoc test and one-way analysis of variance (ANOVA) were used for multiple comparisons. The standard error of the mean (SEM) was used to show data variability. A significance level of p≤0.05 was employed for statistical significance. The severity of atherosclerotic lesions was measured and described using the median and interquartile range. The Kruskall-Wallis test was used to determine the statistical significance of the median difference of a quantitative non-normally distributed parameter among more than two groups, while the Mann-Whitney test was used to assess the statistical significance of the variance in the median between two groups.

### Tissue sample preparation

Aortic arches were externalized, cleared of connective tissue and adhering fat, and divided into two pieces. One piece of aortic tissue was immediately immersed in a 10% formaldehyde solution for 24 hours to create histopathology and immunostaining slides. The second piece of aortic tissue was homogenized in TRIzol^®^ reagent to assess the NLRP3 inflammasome marker using the RT-PCR method.

## RESULTS

### Effects of atherogenic diet and therapy on NLRP3 inflammasome pathway parameter mRNA expression

**Figure 1:**
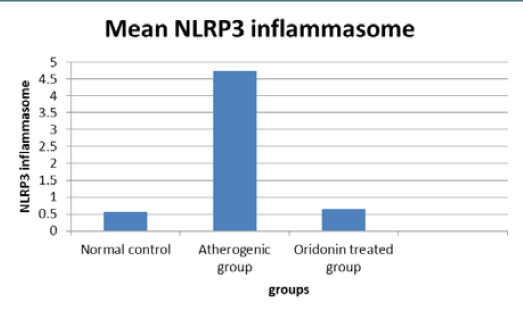
The difference in mean NLRP3 inflammasome among the three study groups

The average expression of the NLRP3 inflammasome mRNA was lowest in animals on a normal diet (0.56) and greatest in animals on an atherogenic diet 4.74, as shown in Figure 1. Compared to animals on a normal diet, an atherogenic diet was linked with a statistically significant (p≤0.001) increase in the mean expression of the NLRP3 inflammasome mRNA. The Oridonin-treated group showed a statistically significant (p≤0.001) decline in the mean expression of NLRP3 inflammasome mRNA compared to the atherogenic group.

### Impact of atherogenic diet and Oridonin on IL-1B immunostaining intensity

**Figure 2:**
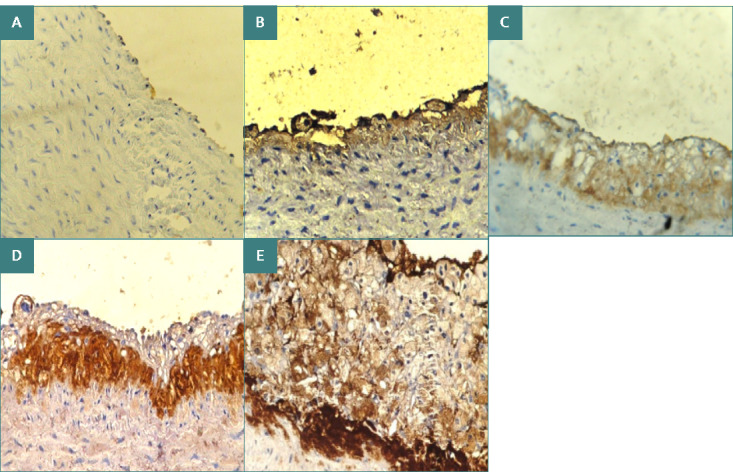
Immunohistochemical staining of IL-1B expression in aortic arches of cholesterol-fed rabbits (x40). A: negative, B: weak stain intensity, C: moderate stain intensity, D: strong stain intensity, E: very strong stain intensity

As shown in Figure 2 and Table 2, animals in the normal control group (negative) had the lowest median intensity of IL-1B immunostaining, while animals in the atherogenic control group had the highest intensity (++++). The atherogenic group had significantly higher mean rank of IL-1B immunostaining compared to the normal control group (mean rank 25, 4 respectively; p≤0.001). In contrast, the Oridonin-treated group showed significantly lower IL-1B immunostaining intensity than the atherogenic group (mean rank 14.5, 25 respectively; p≤0.001).

**Table 2:** The difference in median IL-1B immunostaining intensity among the study groups

	Normal control group		Atherogenic group		Oridonin treated group	
	N	%	N	%	N	%
Immunohistochemistry (IL-1B)						
Negative	7	100	0	0	0	0
+	0	0	0	0	5	71.4
++	0	0	0	0	2	28.6
+++	0	0	2	28.6	0	0
++++	0	0	5	71.4	0	0
Total	7	100	7	100	7	100
Median	Negative		++++		++	
Mean rank	4		25		14.5	
p (Mann-Whitney) for the difference in the median between 2 groups:						
Atherogenic group X Normal control group=0.001						
Oridonin X Atherogenic group=0.001						

### Severity of aortic atherosclerosis in different study groups

**Figure 3:**
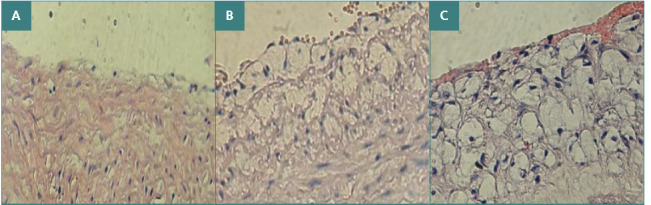
A cross-section of a hypercholesterolemia rabbit's aortic arch showed the progression of atherosclerosis (x40). A: normal arterial morphology in the control group; B: early atherosclerotic lesion in the group treated with Oridonin; C: advanced atherosclerotic lesion in the atherogenic group

The median degree of aortic atherosclerotic lesions was highest in the control animals fed an atherogenic (advanced) diet and lowest in the normal control group fed a normal diet (Figure 3 and Table 3). The atherogenic group had a statistically significant (p≤0.001) higher median atherosclerotic lesion degree when compared to the healthy control group. The initial atherosclerotic lesion in the group treated with Oridonin was statistically (p≤0.001) less severe compared to the atherogenic group.

**Table 3:** The difference in median atherosclerotic lesion degree among the three study groups

	Normal Diet Control Group	Atherogenic Diet Control Group	Oridonin Treated Group	p-value (Kruskal- Wallis)
Aortic atherosclerotic lesion severity				<0.001
Mean rank	4	25	14	

Additionally, post-hoc Mann-Whitney tests were performed to assess specific pairwise comparisons between the study groups. The results indicated significant differences between the atherogenic diet control group and the normal control group (p=0.001), as well as between the Oridonin-treated group and the atherogenic control group (p=0.001) and the Oridonin-treated group and the normal control group (p=0.001).

## DISCUSSION

The results of our study revealed a significant increase in NLRP3 inflammasome mRNA expression among animals on an atherogenic diet, which is consistent with previous studies conducted by Xintong *et al*. [17] and Xinxu *et al*. [18]. These studies demonstrated that cholesterol crystals and oxidized low-density lipoprotein (LDL) act as danger signals, activating the NLRP3 inflammasome in macrophages. This activation leads to caspase-1-mediated maturation and secretion of IL-1β, further promoting the inflammatory response associated with atherosclerosis [17, 18].

Conversely, the Oridonin-treated group exhibited a significant decrease in NLRP3 inflammasome mRNA expression, supporting the findings of Hongbin *et al*. [19] and Chuanghong Lu *et al*. [20].

Ori is a specific and covalent inhibitor for NLRP3 inflammasome. Oridonin is known to be a specific and covalent inhibitor for the NLRP3 inflammasome. Our analysis demonstrated that rabbits in the atherogenic control group displayed high immunostaining intensity for IL-1B, supporting similar findings in studies by Yina Yoon *et al*. and Jan Hettwer *et al*. [21, 22]. Cholesterol crystals present in plaques have the ability to stimulate the NACHT, LRR, and PYD domains-containing protein 3 (NLRP3) inflammasome in macrophages, resulting in the secretion of mature interleukin-1b [23]. Ori treated group showed significantly lower IL-1B immunostaining intensity (+) than the atherogenic group. Liang *et al*. and Chunyan Li *et al*. showed that Oridonin reduced the level of IL-1β by blocking the interaction between NLRP3 and NEK7 [24, 25]. Oridonin acts as a specific and covalent inhibitor for the NLRP3 inflammasome by forming a covalent bond with cysteine 279 of the NLRP3 in the NACHT domain. This interaction blocks the association between NLRP3 and NEK7, ultimately inhibiting NLRP3 inflammasome assembly and activation. Furthermore, Oridonin inhibits caspase-1 activation, preventing the cleavage of pro-IL-1β and pro-IL-18 and reducing the production of mature and functional IL-1β and IL-18 [19].

## CONCLUSION

The results of this study demonstrate that the administration of Oridonin led to a significant reduction in the severity of initial atherosclerotic lesions compared to the atherogenic group. This beneficial effect of Oridonin is likely attributed to its specific inhibition of the NLRP3 inflammasome pathway.

## Personal thanks

The authors express their gratitude to the members of the Pharmacology and Therapeutic Department at Kufa Medical College for their valuable support and contributions to this study.
